# On a Novel Hybrid Manta Ray Foraging Optimizer and Its Application on Parameters Estimation of Lithium-Ion Battery

**DOI:** 10.1007/s44196-022-00114-4

**Published:** 2022-08-10

**Authors:** Rizk M. Rizk-Allah, Mohamed I. Zineldin, Abd Allah A. Mousa, S. Abdel-Khalek, Mohamed S. Mohamed, Václav Snášel

**Affiliations:** 1 Basic Engineering Science Department, Faculty of Engineering, Menoufia University, Shebin El-Kom, 32511 Egypt; 2 Faculty of Engineering, Menoufia University, Shebin El-Kom, Egypt; 3 Department of Mathematics, College of Science, Taif University, Taif, 21944 Saudi Arabia; 4 Faculty of Electrical Engineering and Computer Science, VŠB-Technical University of Ostrava, Poruba, 70800 Ostrava, Czech Republic

**Keywords:** Meta-heuristic algorithm, Manta ray foraging optimization, Particle swarm optimization, Swarm optimization, Tremblay's model, Li-ion battery, Battery dynamics model

## Abstract

In this paper, we propose a hybrid meta-heuristic algorithm called MRFO-PSO that hybridizes the Manta ray foraging optimization (MRFO) and particle swarm optimization (PSO) with the aim to balance the exploration and exploitation abilities. In the MRFO-PSO, the concept of velocity of the PSO is incorporated to guide the searching process of the MRFO, where the velocity is updated by the first best and the second-best solutions. By this integration, the balancing issue between the exploration phase and exploitation ability has been further improved. To illustrate the robustness and effectiveness of the MRFO-PSO, it is tested on 23 benchmark equations and it is applied to estimate the parameters of Tremblay's model with three different commercial lithium-ion batteries including the Samsung Cylindrical ICR18650-22 lithium-ion rechargeable battery, Tenergy 30209 prismatic cell, Ultralife UBBL03 (type LI-7) rechargeable battery. The study contribution exclusively utilizes hybrid machine learning-based tuning for Tremblay's model parameters to overcome the disadvantages of human-based tuning. In addition, the comparisons of the MRFO-PSO with six recent meta-heuristic methods are performed in terms of some statistical metrics and Wilcoxon’s test-based non-parametric test. As a result, the conducted performance measures have confirmed the competitive results as well as the superiority of the proposed MRFO-PSO.

## Introduction

To solve hard and complicated real engineering problems, engineers should take the right decisions variables, and then, they will need a vital process for attaining the best solution which is named optimization. Therefore numerous methods were presented to solve optimization problems particularly nonlinear problems (NLPs), some of them were conventional and others were known as metaheuristics. Meta-heuristic algorithms (MetAs) are powerful artificial intelligence tools that can be classified to subcategories: chemical-based optimization algorithms like equilibrium optimizer (EO) [1], the chemical reaction based optimization algorithm [2] bio-inspired methods like coronavirus optimization algorithm [3], co-evolving algorithms [4], quantum evolutionary algorithm [5], quantum-inspired acromyrmex evolutionary algorithm [6], genetic algorithm (GA) [7], tabu search [8], cultural algorithm [9], stochastic fractal search [10], backtracking optimization algorithm [11], biogeography-based optimization algorithm (BBO) [12], swarm intelligence methods such as artificial immune system [13], memetic algorithm [14], group search optimizer [15], beehive algorithm [16], wolf search algorithm [17], Egyptian vulture optimization algorithm [18], swallow swarm optimization algorithm [19], ant lion algorithm (ALO) [20], grey wolf optimization (GWO) [21], chicken swarm optimization [22], shark smell optimization [23], butterfly-inspired algorithm [24], physics-based methods like black hole (BH) [25], simulated annealing (SA) [26], lightning search algorithm (LSA) [27], water cycle process (WCP) [28], multiple cyclic swarming optimization [29], colliding bodies optimization (CBO) [30], behavior-based techniques like brain storm optimization [31], volleyball premier league algorithm [32], gaining-sharing knowledge based algorithm [33], teaching-learning-based optimization (TLBO) [34], league championship algorithm (LCA) [35], mine blast algorithm (MBA) [36], flower pollination algorithm (FPA) [37, 38], trigonometric-based like sine–cosine algorithm (SCA) [39], etc. Additionally, researchers chose another pursuit through combining some properties of two or more techniques to improve efficiency and shorten the computational time. In this regard, some several attentions have been developed such as PSO-GA hybrid with Adam optimization [40], a synergy of the sine–cosine algorithm and particle swarm optimizer (SCA-PSO) [41], hybrid sine–cosine algorithm with differential evolution (SCA-DE) [42], hybrid DE and extremal optimization (DE-EO) [43], hybrid fruit fly optimization algorithm and firefly algorithm (FOA-FA) [44], hybrid Grey wolf optimization with particle swarm optimization (GWO-PSO) [45], enhanced tunicate swarm algorithm (ETSA) [46], hybrid ABC, and PSO [47]. The traditional methods with their two forms, direct and gradient-based methods, face some serious disadvantages for example, the delay in direct search methods or non-differentiability and discontinuity in gradient-based methods. Also, they rely on the initial solution and may fail to reach the promising regions. On the other hand, metaheuristics have proven their worth as they overcome the previous shortages of traditional methods. Meta-heuristic algorithms are suitable for non-convex, non-differentiable or discontinuous fitness functions and constraints. In addition, they can avoid being trapped in local optima in sharp and multiple peak problems. Moreover, they avoid computation of the gradients of the objective function and the constraints as well [44]. Lately, MRFO has gained popularity, since it is deployed in many engineering and other fields for example, Alturki et al. presented an MRFO-based optimal control strategy to enhance the proportional-integral (PI) controllers of DC/DC and DC/AC converters for PV grid-connected system [48]. Jinlin Wei et al. proposed filtering equipment protection based on MRFO, which improves the internal capacitance distribution of filtering device. To attain this goal, the unbalanced current generated due to the alerted capacitance should be minimized to keep the device safe [49]. Ouyang et al. used MRFO to determine the K-means’ initial center of clustering, which optimized the image segmentation efficiency [50]. Chattopadhyay et al. deployed an MRFO in feature selection for recognizing speech emotion, which increased the classification accuracy significantly [51]. Tiwari et al. minimized the total operating cost for distributed generator evaluated by load dispatch [52], while Sultan et al. used MRFO to solve multi-objective problems of sizing components of hybrid PV, wind turbine, and fuel cell system [53]. Simultaneously, other researchers have integrated MRFO with other algorithms like, Duan et al. replaced the clan updating operator in the elephant herding optimization (EHO) method with the somersault foraging tactic of Manta rays, and enhanced the diversity of the population by the Gaussian mutation [54]. Houssein et al. [55] proposed that a modified MRFO with opposition-based learning (OBL), named MRFO-OBL, was employed to solve the problem of the image segmentation with multilevel thresholding’s, where the MRFO-OBL was employed to identify the COVID-19 using chest CT images. Houssein et al. [56] applied the MRFO to optimize the parameters of support vector machine (SVM) to classify the electrocardiogram (ECG) arrhythmia. In addition, Karuppusamy proposed a hybrid MRFO for feature selection and Convolutional Neural Network (CNN) as classifier for brain tumor detection [57]. An improved version of the MRFO based on Levy flight and Morlet wavelet mutation strategy for extracting the Magnetorheological (MR) dampers control parameters has been proposed. This version was tested on CEC 2014 and CEC 2017 benchmark problems [58]. While Abdul Razak et al. adopted the GA’s mutation and crossover to improve MRFO’s convergence action, where the proposed genetic MRFO (GMRFO) was optimized an interval type 2 fuzzy logic for inverted pendulum system [59]. Also, GMRFO was tested on some composite natures of the test functions. Quantum MRFO (QMRFO) has been proposed by Ramadan et al. to estimate the parameters of the three diode solar photovoltaic model [60]. In addition, a gradient-based optimizer (GBO) hybridized with MRFO, named MRFO–GBO, has been solved the multi-objective economic emission dispatch (EED) problems [61].

Despite the fact that the aforementioned (MetAs) have been presented their abilities while dealing with different optimization issues; however, because of the no-free-lunch theorem [62], there is potential attempt to investigate different algorithms for further improvement when dealing with some optimization tasks. The revelation that the NFL theorem exists has encouraged this work to improve MRFO's capabilities by developing a hybrid variant with the PSO algorithm. Therefore, this paper presents a hybrid variant of the Manta ray foraging optimization (MRFO) with the particle swarm optimization (PSO), named MRFO-PSO, to achieve better balance among the exploration and exploitation abilities. The performance of the MRFO-PSO is validated on 23 benchmark problems and its applicability is confirmed through estimating the parameters of Tremblay's model with three different commercial lithium-ion batteries. The statistical measures along with pairwise tool have affirmed that the MRFO-PSO is capable of realizing very promising performances when compared with other optimizers.

The reminder sections of this paper is arranged as follows. In Sect. 2, material and methods regarding the mathematical representation of the function optimization and basics of the original MRFO and PSO. The producers of the proposed MRFO-PSO are presented in Sect. 3. In Sect. 4, the experimental simulations and results regarding the function optimization and lithium-ion battery are presented. In Sect. 5, the findings are concluded.

## Materials and Methods

### The Mathematical Statement of the Optimization Problem

Generally, any optimization problem has a standard formulation as follows.

1 $$\begin{array}{l}Min\,F(x),\\ x\in {\mathfrak{R}}^{n}\mid x=\left({x}_{1},{x}_{2},{x}_{3},\dots {x}_{n}\right)\\ {l}_{i}\le {x}_{i}\le {u}_{i},i=\mathrm{1,2},\dots ,n,\end{array},$$

where $F\left({\varvec{x}}\right)$ is the objective or fitness function, which should be minimized for design space ${\mathfrak{R}}^{n}$ (${\mathfrak{R}}^{n}$ defines the set of all ordered $n$-tuples of real numbers), in which there are $n$ dimensions of candidate solutions usually called feasible solutions, ${x}_{i}$ denotes the *i*th element of the decision vector (${\varvec{x}}),$ and ${u}_{i}$ and ${l}_{i}$ define the lower and upper limits, respectively.

### Basics of MRFO

MRFO was proposed in 2020 by Zhao et.al [63] based on the foraging strategy of giant marine creatures called Manta rays which have a bird shape-like. It initializes a population of candidate solutions which act as Manta rays individuals searching for the best position. The plankton is consumed to be concentrated; also the best solution obtained so far acts as the plankton. The search strategy consists of three phases: chain foraging, cyclone foraging, and somersault foraging.

#### Chain Foraging Phase

In this phase, every fish in Manta rays' school follows its frontal individual moving in a foraging chain and the best solution found so far. The updating by the chain foraging is formulated mathematically as follows:

2 $$x_{i}^{t + 1} = \left\{ {\begin{array}{*{20}l} {x_{i}^{t} + r\left( {x_{b } - x_{i}^{t} } \right) + a\left( {x_{b } - x_{i}^{t} } \right) \to i = 1} \\ {x_{i}^{t} + r\left( {x_{i - 1}^{t} - x_{i}^{t} } \right) + a\left( {x_{b } - x_{i}^{t} } \right) \to i = 2, \ldots ,N} \\ \end{array} } \right.$$

3 $${\text{a}}=2\mathrm{r}\sqrt{\left|\mathrm{log}(\mathrm{r})\right|,}$$

where $${x}_{i}^{t}$$ represent the $$i$$th individual's position at the iteration ($$t) , r$$ is a random vector belong to [0, 1], $${\text{a}}$$ is weighting function, and $${x}_{b}$$ represents the best position obtained so far. The updated position ($${x}_{i}^{t+1})$$ is performed by the current position ($${x}_{i}^{t})$$ and previous position $${(x}_{i-1}^{t}$$) and the best position$${x}_{b}$$.

#### Cyclone Foraging

Manta ray individuals create a foraging chain along with making spiral movements while searching for the food source. Flocked Manta rays in this step not only follow the Manta ray that in front of the chain but also chase a spiral pattern to get closer to the prey. This spiral movement of the Manta ray in behavior in $n$ dimensional search space is modeled mathematically as follows:

4 $$x_{i}^{t + 1} = \left\{ {\begin{array}{*{20}l} {x_{b} + r\left( {x_{b} - x_{i}^{t} } \right) + B \cdot \left( {x_{b} - x_{i}^{t} } \right) \to i = 1} \\ {x_{b} + r\left( {x_{i - 1}^{t} - x_{i}^{t} } \right) + B \cdot \left( {x_{b} - x_{i}^{t} } \right) \to i = 2, \ldots ,N} \\ \end{array} } \right.$$

5 $$B = 2\exp \left( {r_{1} \cdot \frac{T - t + 1}{T}} \right)*\sin \left( {2\pi r_{1} } \right),$$

where $B$ is weight coefficient, $T$ is the total number of iterations, and ${\text{r}},$ $r_{1}$ $\in \left[ {0,1} \right]$ represent random numbers. The cyclone foraging enables the individuals of Manta rays to exploit the feasible region with the best solution obtained so far. Moreover, for a good exploration, each individual is forced to find a new position globally placed far from its current position by assigning reference position which determined randomly in the whole space. This exploration mechanism is written mathematically as

6 $$x_{i}^{t + 1} = \left\{ {\begin{array}{*{20}l} {x_{{{\text{rand}}}} + r\left( {x_{{{\text{rand}}}} - x_{i}^{t} } \right) + B \cdot \left( {x_{{{\text{rand}}}} - x_{i}^{t} } \right) \to i = 1} \\ {x_{{{\text{rand}}}} + r\left( {x_{i - 1}^{t} - x_{i}^{t} } \right) + B \cdot \left( {x_{{{\text{rand}}}} - x_{i}^{t} } \right) \to i = 2, \ldots ,N} \\ \end{array} } \right.$$

7 $$x_{{{\text{rand}}}} = l_{i} + r^{*} \left( {u_{i} - l_{i} } \right),$$

where ${x}_{\text{rand}}$ is a random position placed indiscriminately in the search space limited by lower and upper bounds ${u}_{i}$ and ${l}_{i}$, respectively.

#### Somersault Foraging

All Manta rays’ individuals swim forward and backward to the pivot with updating their positions by somersaults around the best position obtained so far which are modeled as follows:

8 $${x}_{i}^{t+1}={x}_{i}^{t}+\psi \left({r}_{2}{x}_{b}-{r}_{3}{x}_{i}^{t}\right)\to i=1,\dots ,N,$$

where $\psi$, called somersault factor, it determines the range of somersault in which Manta ray can swim ($\psi =2$), ${r}_{2},{r}_{3}$ are random values within the [0, 1] range. Therefore, the behavior of somersault foraging enables Manta rays to move freely in new domains among their positions and symmetrical positions according to the best position obtained up till now. Also, the somersault range is proportional to iteration inversely; because it is reduced when iteration increases.

### Basics of PSO

Although PSO was proposed in 1995 by Kennedy and Eberhart [64], it has wide popularity in the optimization field due to its superior performance. PSO was inspired by bird flocks while searching their food, PSO starts with a population with $N$ birds which act as feasible solutions, each bird or particle has initial position and velocity. Every bird updates its velocity ${v}_{i}$ as well as its position ${x}_{i}$ in the new iteration $t+1$ considering the personal best position $({P}_{i})$, and the global best position of the whole swarm ($\gamma$) as follows:

9 $$v_{i}^{t + 1} = \omega v_{i}^{t} + c_{1} {\text{rand}} \cdot \left( {P_{i} - x_{i}^{t} } \right) + c_{2} \cdot {\text{rand}} \cdot \left( {\gamma - x_{i}^{t} } \right)$$

10 $$x_{i}^{t + 1} = x_{i}^{t} + v_{i}^{t + 1} ,$$

where ${v}_{i}^{t}, {v}_{i}^{t+1}$ are the particle velocity at the current and next iterations, respectively, $\omega$ is a weighting function $\omega \in \left[\mathrm{0,1}\right]$, ${c}_{1},{c}_{2}$ are weighting constants, $\mathrm{rand}$ is a random number between 0 and 1, and ${x}_{i}^{t+1}, {x}_{i}^{t}$ are the particle position at the current and next iterations, respectively.

## The Proposed MRFO-PSO

**Fig. 1:**
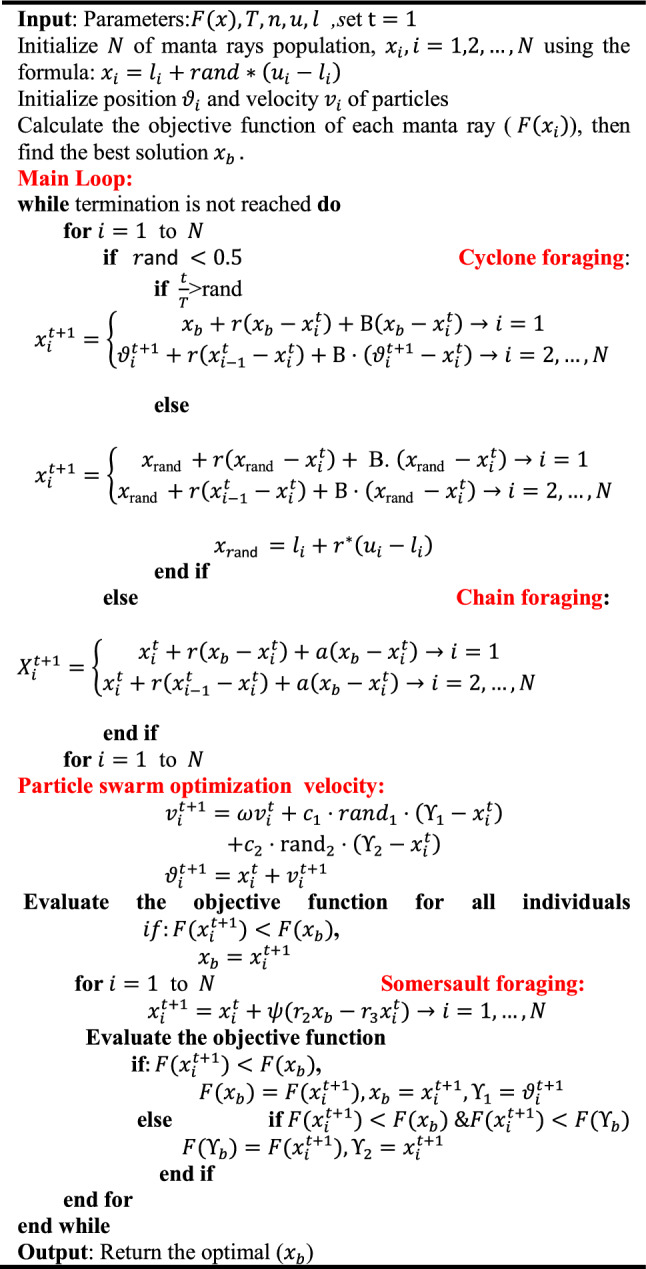
Pseudocode of MRFO-PSO algorithm

**Fig. 2:**
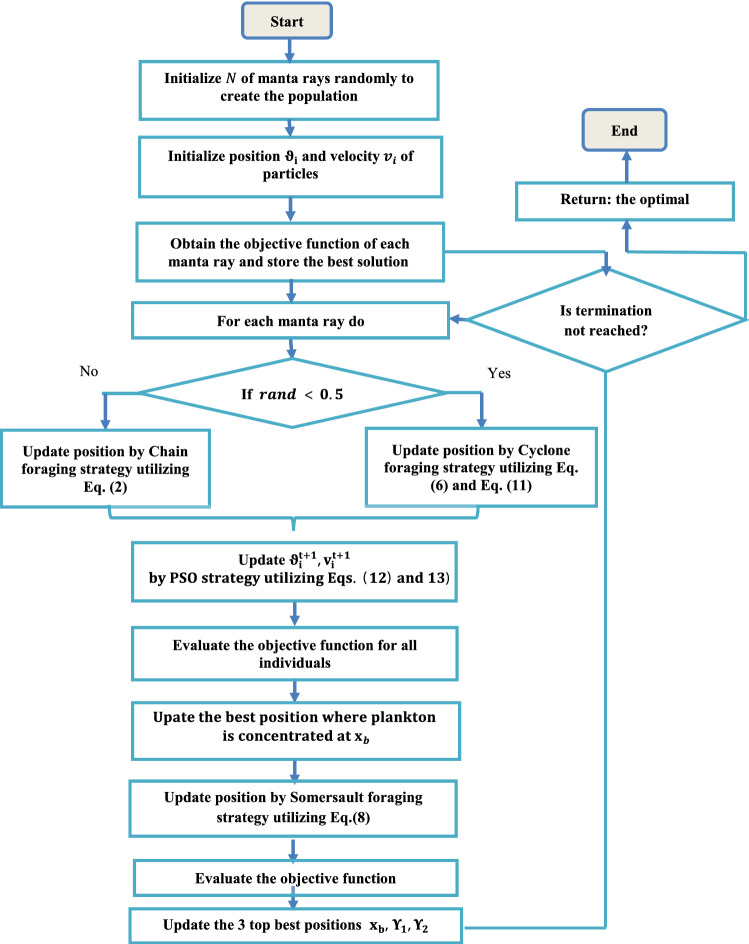
The flowchart of the MRFO-PSO algorithm

In this section, the proposed synergy of the MRFO with PSO is introduced. MRFO and PSO are typical examples of meta-heuristic algorithms and have been employed to deal with various engineering tasks efficiently. However, MRFO lacks for memory to keep the best information of the previous trials. Thus, as MRFO has trouble in reaching a global area, a PSO’s group can boost the searching of the MRFO for attaining optimal seeking system. In this context, the Manta ray individual’s ability is enhanced by utilizing the velocity concept that inspired from PSO in the cyclone foraging phase to update the position of MRFO, where Eqs. (8), and (5) can be modified as follows:

11 $$x_{i}^{{t + 1}} = \left\{ {\begin{array}{*{20}c} {x_{b} + r\left( {x_{b} - x_{i}^{t} } \right) + B \cdot \left( {x_{b} - x_{i}^{t} } \right) \to i = 1} \\ {\vartheta _{i}^{{t + 1}} + r\left( {x_{{i - 1}}^{t} - x_{i}^{t} } \right) + B \cdot \left( {\vartheta _{i}^{{t + 1}} - x_{i}^{t} } \right) \to i = 2, \ldots ,N} \\ \end{array} } \right.$$

12 $$v_{i}^{t + 1} = \omega v_{i}^{t} + c_{1} {\text{rand}}_{1} \cdot \left( {\Upsilon_{1} - x_{i}^{t} } \right) + c_{2} \cdot {\text{rand}}_{2} \cdot \left( {\Upsilon_{2} - x_{i}^{t} } \right)$$

13 $$\vartheta_{i}^{t + 1} = x_{i}^{t} + v_{i}^{t + 1} ,$$

where ${\vartheta }_{i}^{t+1}$ is the individual position reached by its velocity ${v}_{i}^{t+1}$, and ${\Upsilon }_{1}$ is the best position reached by its velocity obtained so far, while ${\Upsilon }_{2}$ is the second-best individual position reached in the last iteration. Besides, the pseudo-code of the MRFO-PSO algorithm is shown in Fig. 1, while the flowchart is shown in Fig. 2.

## Results and Discussion

In this section, we tested the performance of the MRFO-PSO on 23 benchmark functions and utilized our hybrid algorithm to extract parameters of three cases of Li-ion batteries. The experiments are conducted on Matlab 2013a with device specifications: Processor Intel® core ™ i7-7500U CPU@ 2.70 GHz 2.90GHZ. RAM 8 GB and 64-bit operating system.

### Benchmark Functions

To prove the effectiveness of the MRFO-PSO, it is tested the on different natures of benchmark problems such as Kowalik's, Goldstein-Price's, Foxholes's, Branin's, HGBat's, Rastrigin's, and Schwefel's functions. These functions have an assortment of difficult obstacles regarding the objective function such as noise, rotation, ill-conditioning, multimodality, and non-separable. We considered the parameter settings of algorithms as suggested in the corresponding literature. Tables 1, 2, 3 illuminate the 23 test functions and their peculiarities, formularizations, dimensions ($n$), range ($[{l}_{i},{u}_{i}], i=\mathrm{1,2},\dots ,n$), and the minimum solution. However, Table 1 shows unimodal functions (F1–F7), Table 2 shows multimodal benchmark functions (F8–F13), and Table 3 shows multimodal benchmark functions with fixed dimension (F14–F23)) with settled dimensions ($n$). The outputs are illustrated in Table 4 which depicts the prior efficiency of MRFO-PSO.

**Table 1:** Unimodal benchmark functions

Function name	Formula	*n*	Range	Minimum
Sphere	${F}_{1}={\sum }_{i=1}^{n}{x}_{i}^{2}$	100	[− 100, 100]	0
Schwefel 2.22	${F}_{2}={\sum }_{i=1}^{n}|{x}_{i}|+{\prod }_{i=1}^{n}|{x}_{i}|$	100	[− 10, 10]	0
Schwefel 1.2	${F}_{3}={\sum }_{i=1}^{n}{\left({\sum }_{j-1}^{i}{x}_{j}\right)}^{2}$	100	[− 100, 100]	0
Schwefel 2.21	${F}_{4}={\mathit{max}}_{i}\{|{x}_{i}|,1\le i\le n\}$	100	[− 100, 100]	0
Rosenbrock	${F}_{5}={\sum }_{i=1}^{n-1}[100({x}_{i+1}-{x}_{i}^{2}{)}^{2}+({x}_{i}-1{)}^{2}]$	100	[− 30, 30]	0
Step	${F}_{6}={\sum }_{i=1}^{n}([{x}_{i}+0.5]{)}^{2}$	100	[− 100, 100]	0
Quartic	${F}_{7}={\sum }_{i=1}^{n}i{x}_{i}^{4}+random[\mathrm{0,1})$	100	[− 1.28, 1.28]	0

**Table 2:** Multimodal benchmark functions

Function name	Formula	*n*	Range	Minimum
Schwefel	${F}_{8}={\sum }_{i=1}^{n}-{x}_{i}\mathit{sin}(\sqrt{|{x}_{i}|})$	100	[− 500, 500]	− 418.9829*5
Rastrigin	${F}_{9}={\sum }_{i=1}^{n}[{x}_{i}^{2}-10\mathit{cos}(2\pi {x}_{i})+10]$	100	[− 5.12, 5.12]	0
Ackley	${F}_{10}=-20\mathit{exp}(-0.2\sqrt{\frac{1}{n}{\sum }_{i=1}^{n}{x}_{i}^{2}})-\mathit{exp}(\frac{1}{n}{\sum }_{i=1}^{n}\mathit{cos}(2\pi {x}_{i}))+20+e$	100	[− 32, 32]	0
Griewank	${F}_{11}=\frac{1}{4000}{\sum }_{i=1}^{n}{x}_{i}^{2}-{\prod }_{1=1}^{n}\mathit{cos}(\frac{{x}_{i}}{\sqrt{i}})+1$	100	[− 600,600]	0
Penalized	$$F_{12} = \frac{\pi }{n}\left\{ {10\sin (\pi y_{i} ) + \mathop \sum \limits_{i = 1}^{n - 1} (y_{i} - 1)^{2} \left[ {1 + 10\sin^{2} (\pi y_{i + 1} )} \right] + (y_{n} - 1)^{2} } \right\} + \mathop \sum \limits_{i = 1}^{n} u\left( {x_{i} ,10,100,4} \right)$$$$y_{i} = 1 + \frac{{x_{i} + 1}}{4}u\left( {x_{i} ,a,k,m} \right) = \left\{ {\begin{array}{*{20}l} {k(x_{i} - a)^{m} x_{i} > a} \\ {0 - a < x_{i} < a} \\ {k( - x_{i} - a)^{m} x_{i} < - a} \\ \end{array} } \right.$$	100	[− 50, 50]	0
Penalize 2	$$\begin{aligned} F_{13} & = 0.1\left\{ {\sin^{2} (3\pi x_{1} ) + \mathop \sum \limits_{i = 1}^{n} (x_{i} - 1)^{2} \left\{ {1 + \sin^{2} (3\pi x_{i} + 1)} \right\} + \left( {x_{n} - 1)^{2} \left[ {1 + \sin^{2} (2\pi x_{n} } \right)} \right]} \right\} \\ & + \mathop \sum \limits_{i = 1}^{n} u\left( {x_{i} ,5,100,4} \right) \\ \end{aligned}$$	30	[− 50, 50]	0

**Table 3:** Multimodal benchmark functions with fixed dimension

Function name	Formula	*n*	Range	Maximum
Foxholes	${F}_{14}={\left(\frac{1}{500}+{\sum }_{j=1}^{25}\frac{1}{j+{\sum }_{i=1}^{2}({x}_{i}-{a}_{ij}{)}^{6}}\right)}^{-1}$	2	[− 65, 65]	1
Kowalik	${F}_{15}={{\sum }_{i=1}^{11}\left[{a}_{i}-\frac{{x}_{1}({b}_{i}^{2}+{b}_{i}{x}_{2})}{({b}_{i}^{2}+{b}_{i}{x}_{3}+{x}_{4})}\right]}^{2}$	4	[− 5, 5]	0.00030
Six-hump Camel-Back	${F}_{16}=4{x}_{1}^{2}-2.1{x}_{1}^{4}+\frac{1}{3}{x}_{1}^{6}+{x}_{1}{x}_{2}-4{x}_{2}^{2}+4{x}_{2}^{4}$	2	[− 5, 5]	− 1.0316
Branin	${F}_{17}=({x}_{2}-\frac{5.1}{4{\pi }^{2}}{x}_{1}^{2}+\frac{5}{\pi }{x}_{1}-6{)}^{2}+10(1-\frac{1}{8\pi })\mathit{cos}{x}_{1}+10$	2	[− 5, 5]	0.398
Goldstein-Price	$F_{18} = \left[ {1 + \left( {x_{1} + x_{2} + 1} \right)^{2} \left( {19 - 14x_{1} + 3x_{1}^{2} - 14x_{2} + 6x_{1} x_{2} + 3x_{2}^{2} } \right)} \right] + \left[ {30 + \left( {2x_{1} - 3x_{2} } \right)^{2} \left( {18 - 32x_{1} + 12x_{1}^{2} + 48x_{2} - 36x_{1} x_{2} + 27x_{2}^{2} } \right)} \right]$	2	[− 2, 2]	3
Hartman 3	${F}_{19}=-{\sum }_{i=1}^{4}{c}_{i}\mathit{exp}(-{\sum }_{j=1}^{3}{a}_{ij}({x}_{j}-{p}_{ij}{)}^{2})$	3	[1, 3]	− 3.86
Hartman 6	${F}_{20}=-{\sum }_{i=1}^{4}{c}_{i}\mathit{exp}(-{\sum }_{j=1}^{6}{a}_{ij}({x}_{j}-{p}_{ij}{)}^{2})$	6	[0, 1]	− 3.32
Shekel5	${F}_{21}=-{\sum }_{i=1}^{5}[(X-{a}_{i})(X-{a}_{i}{)}^{T}+{c}_{i}{]}^{-1}$	4	[0, 10]	− 10.1532
Shekel7	${F}_{22}=-{\sum }_{i=1}^{7}[(X-{a}_{i})(X-{a}_{i}{)}^{T}+{c}_{i}{]}^{-1}$	4	[0, 10]	− 10.4028
Shekel10	${F}_{23}=-{\sum }_{i=1}^{10}[(X-{a}_{i})(X-{a}_{i}{)}^{T}+{c}_{i}{]}^{-1}$	4	[0, 10]	− 10.5363

**Table 4:** The results obtained by MRFO-PSO and the compared algorithms

Function		MRFO-PSO	MRFO	FFA	WOA	DA	GWO	ALO
F1	Mean	**0**	**0**	3817.449775	2.87433E−88	35,282.67205	1.38286E−18	6087.895432
	Min	**0**	**0**	2105.653131	8.8553E−106	16,983.03329	2.0819E−19	1298.237375
	Median	**0**	**0**	3446.814796	1.21248E−92	37,709.76737	4.59058E−19	6364.63198
	Max	**0**	**0**	5787.94451	2.50323E−87	45,916.68548	5.14917E−18	11,095.4251
	Std	**0**	**0**	1243.886718	7.82369E−88	8554.372278	1.67148E−18	2734.027906
F2	Mean	5.4000e-323	0	609,563.2103	4.55E−73	251.8555159	1.48E−11	275.5777969
	Min	**0**	**0**	119.0660968	7.24E−91	185.0443446	3.23E−12	73.77359442
	Median	4.000e-323	0	242.0847918	5.62E−79	258.8857493	1.50E−11	344.1806061
	Max	1.63000e-322	1.000e-323	3,008,033.994	4.48E−72	327.2339334	3.50E−11	492.7632379
	Std	**0**	0	1,254,201.635	1.42E−72	47.16819385	1.06E−11	164.1384262
F3	Mean	**0**	**0**	14,300.60592	1,275,626.576	386,495.5268	877.3317939	158,447.0097
	Min	**0**	**0**	4403.734738	870,885.9905	155,522.42	12.46037695	107,537.8744
	Median	**0**	**0**	14,203.10034	1,202,193.133	381,827.6966	566.4772898	158,306.3092
	Max	**0**	**0**	23,444.4424	2,186,282.857	610,747.4214	2289.473947	216,821.7413
	Std	**0**	**0**	6260.047332	401,176.9861	141,025.6504	796.7864346	38,326.77051
F4	Mean	**4.9000e−324**	**4.9000e−324**	11.76854073	86.56621715	56.69635796	1.412702635	47.46176646
	Min	**4.9000e−324**	**4.9000e−324**	8.823440783	58.02233218	48.2289067	0.120170667	36.36555695
	Median	**4.9000e−324**	**4.9000e−324**	11.54787131	91.36159745	55.54135234	0.49188892	45.55449513
	Max	**4.9000e−324**	**4.9000e−324**	15.33870828	97.6117535	69.1417825	4.753266729	56.69230437
	Std	**0**	**0**	2.177977245	13.10271064	6.206412802	1.748485093	7.104306705
F5	Mean	**95.7545523**	95.90239626	27,183,858.7	98.51332721	40,937,492.17	98.3860123	3,493,053.094
	Min	**94.56670397**	94.60155512	6,377,308.717	98.41256315	8,920,920.797	97.15001233	683,865.0899
	Median	**95.44217459**	95.42796007	21,295,011.06	98.50675822	48,748,874.87	98.52797034	2,495,141.819
	Max	**98.04207247**	98.08408475	59,845,760.14	98.61784907	64,315,521.53	98.6225629	12,592,833.86
	Std	1.11754043	1.280741261	19,714,404.96	0.059886588	18,867,011.22	0.438844214	3,440,377.293
F6	Mean	**0.4875755**	2.228825765	3053.870864	9.730412689	48,210.65852	13.62691448	5630.525305
	Min	**0.099236103**	0.815721485	1679.634355	6.105723301	38,851.00965	11.49277878	2789.914238
	Median	**0.439872669**	2.342475822	2860.397428	10.04908464	44,515.63681	13.75723631	5177.045267
	Max	**1.281984631**	3.05914942	5060.218761	12.40259428	62,986.08577	15.64263317	9142.217634
	Std	**0.35084462**	0.79398511	1132.487575	1.986438853	8954.835023	1.153952344	1992.286637
F7	Mean	**0.**00022799	0.000167533	390.7854862	0.005953347	96.46098356	0.007489531	8.115407577
	Min	**2.33E−05**	3.88E-05	58.65337143	0.000236507	33.81323153	0.004039786	5.226767194
	Median	0.000212247	0.000132769	446.1443703	0.001699469	89.91059509	0.006330992	8.115958713
	Max	**0.000436223**	0.000490992	589.559281	0.024495723	174.561456	0.01221833	10.84973065
	Std	**0.00012425**	0.000139686	160.2588464	0.008037843	45.44031662	0.003232453	1.743122206
F8	Mean	− 23,724.19594	− 23,165.98707	− 3570.382045	− **33,266.16802**	− 8703.810113	− 15,287.0667	− 18,387.8032
	Min	− 26,351.64017	− 26,338.03233	− 4584.738523	− **41,878.6229**	− 11,103.19629	− 17,622.48337	− 21,347.78934
	Median	− 23,407.55732	− 23,147.82477	− 3534.347237	− **29,816.75778**	− 8978.139541	− 15,295.63798	− 18,058.91585
	Max	− 21,758.43825	− 20,889.16993	− 3063.448067	− **28,168.97945**	− 6085.408901	− 13,807.04388	− 18,058.91585
	Std	1556.74181	**1436.900615**	449.4669363	5841.161477	1401.627779	1126.508375	1040.033117
F9	Mean	**0**	**0**	1108.38547	**0**	918.3722704	4.447724821	449.7071309
	Min	**0**	**0**	897.5395833	**0**	632.6363248	4.55E-13	341.1086913
	Median	**0**	**0**	1102.997006	**0**	925.3340247	2.467509263	435.5323673
	Max	**0**	**0**	1335.509738	**0**	1154.114881	19.1559801	608.9555748
	Std	**0**	**0**	142.8850082	**0**	134.9362478	6.067417029	81.8649115
F10	Mean	**8.88E−16**	**8.88E−16**	7.106917995	3.73E−15	16.50016114	1.17E−10	16.13478871
	Min	**8.88E−16**	**8.88E−16**	4.562451465	**8.88E−16**	15.84470261	3.20E−11	14.23492759
	Median	**8.88E−16**	**8.88E−16**	7.327806968	4.44E−15	16.46211152	9.09E−11	16.59375056
	Max	**8.88E−16**	**8.88E−16**	9.016048676	4.44E−15	17.3617155	3.05E−10	17.19230433
	Std	**0**	**0**	1.327721191	1.50E−15	0.571604928	7.78E−11	0.98408625
F11	Mean	**0**	**0**	1296.662111	**0**	437.7148993	8.88E−17	52.96208371
	Min	**0**	**0**	1259.012386	**0**	297.173223	**0**	18.23072152
	Median	**0**	**0**	1296.563627	**0**	419.9636712	1.11E−16	56.4126613
	Max	**0**	**0**	1347.138349	**0**	681.4814938	3.33E−16	79.90073666
	Std	**0**	**0**	30.66194472	**0**	112.4108763	1.02E−16	19.29865778
F12	Mean	**0.00345953**	0.019507632	7417.011473	0.212839096	24,006,927.5	0.436748212	30,244.63358
	Min	**0.000854047**	0.012361309	10.26618196	0.123313079	7,141,327.08	0.343595105	144.3287168
	Median	**0.002362694**	0.018190002	711.0214255	0.176683096	21,399,814.15	0.423742085	8675.11941
	Max	**0.010269186**	0.027753096	55,245.0115	0.448942998	41,747,194.68	0.555506507	181,825.739
	Std	**0.00305566**	0.006105475	17,441.17979	0.095462571	13,056,599.49	0.065652486	55,976.65127
F13	Mean	**9.89097196**	9.898545218	954,372.2288	5.727602622	120,772,077	7.732802789	1,985,286.179
	Min	**9.887680714**	9.893496834	86,350.66424	4.456461082	34,681,560.22	7.160889561	62,287.29061
	Median	**9.890951885**	9.897622255	835,892.3019	5.512363646	74,057,326.52	7.705682845	1,317,536.099
	Max	**9.89355678**	9.907040031	1,940,117.928	7.299432331	367,062,269.4	8.689560809	6,324,295.519
	Std	**0.00194903**	0.00374923	553,039.6878	1.031800536	103,395,801.3	0.433151717	2,137,666.125
F14	Mean	**0.99800384**	0.998003838	13.32161866	2.669439167	4.966903487	7.925530449	3.86223415
	Min	**0.998003838**	**0.998003838**	0.998004464	**0.998003838**	**0.998003838**	**0.998003838**	1.9920309
	Median	**0.998003838**	**0.998003838**	5.025788487	1.496166649	2.980140503	10.76318067	2.982105157
	Max	**0.998003838**	**0.998003838**	99.03385437	10.76318067	22.90063432	12.67050581	7.873992977
	Std	**1.05E−16**	1.96E−16	30.19048911	2.985516658	6.602352987	4.986349132	2.040263377
F15	Mean	**0.**00054737	**0.000527711**	0.002277392	0.000542221	0.005868206	0.00047299	0.001299779
	Min	**0.000307486**	**0.000307486**	0.00143423	0.000308791	0.000764249	0.000307506	0.000882004
	Median	0.000315771	0.**000307486**	0.002251949	0.000556127	0.002797749	0.000517917	0.001311159
	Max	0.001594051	0.**00159405**	0.004023464	0.000757206	0.021438439	0.000599208	0.001637815
	Std	**0.00046449**	0.000472433	0.00068537	0.000159759	0.007741009	0.000118175	0.000265605
F16	Mean	**− 1.031628453**	**− 1.031628453**	− 0.753672101	− 1.031628451	− 1.031566804	− **1.**0316284	− 1.031628453
	Min	**− 1.031628453**	**− 1.031628453**	− 1.031042958	− 1.031628453	− 1.031627873	− 1.031628449	− 1.031628453
	Median	**− 1.031628453**	**− 1.031628453**	− 0.948881891	− 1.031628453	− 1.031619774	− 1.031628422	− 1.031628453
	Max	**− 1.031628453**	**− 1.031628453**	− 0.063157118	− 1.031628445	− 1.031239604	− 1.031628297	− 1.031628453
	Std	**1.**48E**−16**	**7.40E−17**	0.373800663	3.45E−09	0.000119323	4.42E−08	4.18E−13
F17	Mean	**0.**39788736	**0.397887358**	0.479317847	0.397908988	0.397890239	0.397888283	**0.397887358**
	Min	**0.397887358**	**0.397887358**	0.397888383	0.397887491	0.397887364	0.397887413	**0.397887358**
	Median	**0.397887358**	**0.397887358**	0.398871657	0.397900808	0.397888049	0.397888066	**0.397887358**
	Max	**0.397887358**	**0.397887358**	1.193876748	0.397989363	0.397900481	0.397889621	**0.397887358**
	Std	**0**	**0**	0.251101631	3.03E−05	4.98E−06	8.42E−07	1.01E−13
F18	Mean	**3**	**3**	11.90557403	3.000280919	3.000217282	3.000030188	**3**
	Min	**3**	**3**	3.000678776	3.000001951	3.000000002	3.000001128	**3**
	Median	**3**	**3**	4.089018136	3.00006805	3.000025673	3.000016441	**3**
	Max	**3**	**3**	65.46545639	3.001532561	3.001723275	3.00009187	**3**
	Std	2.34E−15	**1.41E−15**	19.26056387	0.000466474	0.000533516	3.54E−05	1.14E−12
F19	Mean	− **0.3004789**	− 0.300478907	− 0.300478907	− **0.30047891**	− 0.300478907	− 0.300478907	− 0.300478907
	Min	− **0.300478907**	− **0.300478907**	− **0.300478907**	− **0.300478907**	− **0.300478907**	− **0.300478907**	− **0.300478907**
	median	− **0.300478907**	− **0.300478907**	− **0.300478907**	− **0.300478907**	− **0.300478907**	− **0.300478907**	− **0.300478907**
	Max	− **0.300478907**	− **0.300478907**	− **0.300478907**	− **0.300478907**	− **0.300478907**	− **0.300478907**	− **0.300478907**
	Std	**6.**90E**-15**	3.51E-16	**0**	**0**	**0**	**0**	**0**
F20	Mean	− 3.262548611	− **3.298216547**	− 2.831599043	− 3.176940451	− 3.246082859	− **3.**2719839	− 3.285926276
	Min	− **3.321995172**	− **3.321995172**	− 3.123434646	− 3.316555003	− 3.321865686	− 3.321991576	− 3.321995172
	Median	− **3.262548611**	− **3.321995172**	− 2.815247618	− 3.170641978	− 3.30895718	− 3.32197271	− 3.321995172
	Max	− **3.20310205**	− **3.20310205**	− 2.607638931	− 3.094605251	− 3.106929403	− 3.086660823	− 3.199467588
	Std	**0.**06266218	**0.050129742**	0.139874537	0.076924725	0.093480078	0.08545736	0.058084026
F21	Mean	− **6.0747981**	− 5.564997924	− 4.581474305	− 7.85237791	− 6.015645127	− **9.646965221**	− 6.619931042
	Min	− **10.15319968**	− **10.15319968**	− 10.15319875	− 10.14954262	− 10.09773454	− 10.15292015	− **10.15319968**
	Median	− **5.055197729**	− **5.055197729**	− 3.667996012	− 10.09336085	− 5.06672207	− 10.15222594	− **5.055197729**
	Max	− **5.055197729**	− **5.055197729**	− 0.293107471	− 2.607648465	− 2.6282272	− 5.100699776	− 2.682860396
	Std	2.149506357	1.612129768	**3.**62819684	3.019200605	2.933215261	1.**597395001**	3.175086367
F22	Mean	− **7.**2137793	− 5.619198699	− 5.801568732	− 5.068347971	− 6.069470304	− **10.40200819**	− 7.148729246
	Min	− **10.40294057**	− **10.40294057**	− 10.39715332	− 10.40172521	− 10.3962719	− 10.40267472	− 10.**40294057**
	Median	− 5.087671825	− 5.087671825	− 5.064916661	− 5.085418216	− 5.12542205	− **10.40203873**	− 7.765881682
	Max	− 5.087671825	− 5.087671825	− 1.59465723	− 0.90984962	− 1.836695553	− **10.40091025**	− 2.765897328
	Std	2.744792976	1.68083556	**3.36785268**	3.257533961	3.102930995	**0.000582737**	3.518563168
F23	Mean	− 7.703139903	− 7.291652399	− 2.548247012	− 6.562080523	− 5.820304368	− **10.53538456**	− 7.01474945
	Min	− **10.53640982**	− **10.53640982**	− 10.53601567	− 10.52713785	− 10.46541562	− 10.53613262	− 10.53640982
	Median	− 7.832445302	− 5.128480787	− 1.513479836	− 5.128183307	− 5.144060895	− **10.53543506**	− 7.832445302
	Max	− 3.835426803	− 5.128480787	− 0.999106748	− 1.633493299	− 1.67652131	− **10.5344374**	− 1.859480301
	Std	3.011307854	2.792642543	2.88460661	3.415595559	3.427387248	**0.000530934**	3.801603702

Bold values of best results among the comparative algorithms are exhibited

### Simulation Results on the Benchmark Problems

**Fig. 3:**
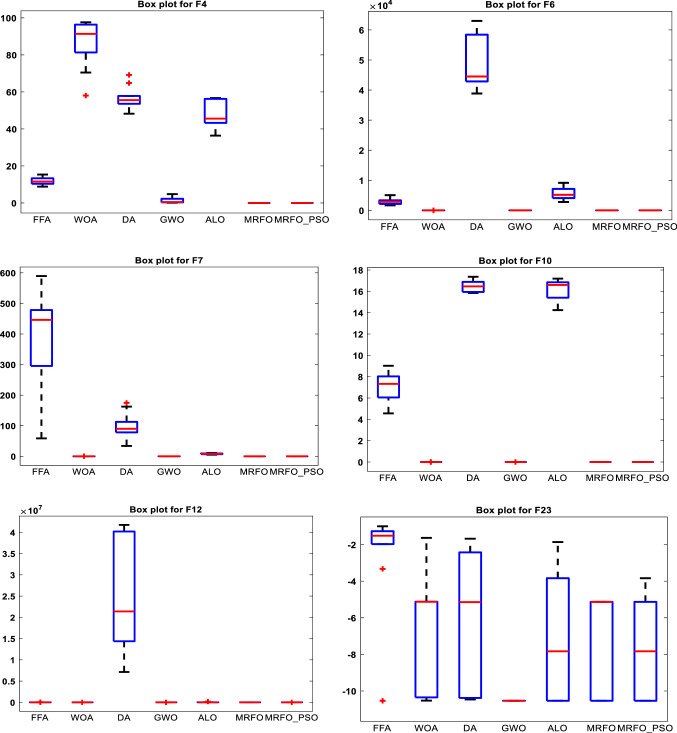
Box plots for some selected functions

**Fig. 4:**
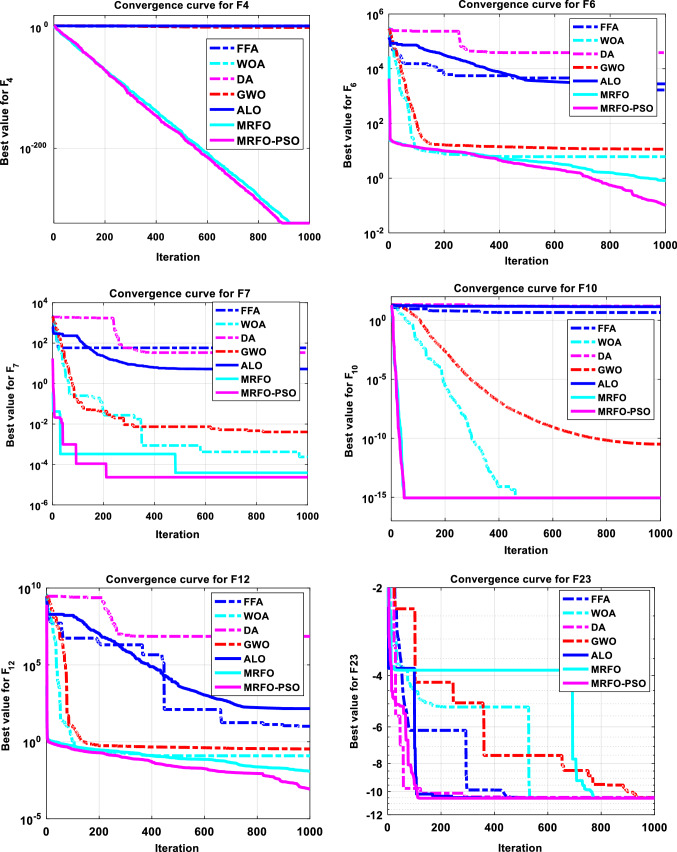
Convergence for some selected results

The performance of the proposed MRFO-PSO is compared with some well-known algorithms include the FFA [44], WOA [65], DA [66], GWO [21], ALO [20], original MRFO, and other state-of-art methods. The obtained outcomes regarding the studied benchmark problems are tabulated in Table 4 using some central tendency statistical metrics which are the average value of the fitness (mean), best value of the fitness (Min), median value (Median), worst value of the fitness (Max), and the standard deviation (STD) to confirm that the archived results are not happen by chance. Based on the mean results, it can be observed that the MRFO-PSO can provide competitive and progressive results in comparison with the other optimizers. These results are obtained through carrying out each algorithm for 30 independent runs. It is found that the MRFO-PSO reaches the optimum for the functions F1, F2, F3, F9, F11, F14, F15, F16, F17, F18, F20, F21, F22, and also overcomes its competitors and gets first in terms of the minimum value in functions F5, F6, F7, F8, F12, F13. In addition, MRFO-PSO gets first in terms of mean, median, maximum, and standard deviation for the functions F1, F3, F4, F6, F9, F10, F11, F12, F13, F14, MRFO-PSO has the least standard deviation for the function F2, F7, F15, F17. MRFO-PSO has the best mean for F5, F16, and F21. MRFO-PSO scores the best median for F16, F17, F18, F19, F20, and F21. MRFO reached the minimum for functions F2, F9, F10. MRFO has the least standard deviation for F8, F16, F20 functions. MRFO gets the least median in functions F14 and F15, F19, F21. MRFO has the best mean for F15 only. MRFO scores the best maximum for functions F14, F15, F19, and F21. While FFA scores least minimum, median, and maximum for F19 only. WOA gets the least mean, median and maximum for F8, WOA gets the least mean, median, maximum, and standard deviation for F9, F11, and F19 functions, where WOA scores the optima in F10, F14. However, DA gets the least min, median, and max in F19 and reaches the minimum for F14. Also, we notice GWO reaches optimum for F11 and F14, F19, but obtain the best median, maximum, and standard deviation for F19, F22, and F23, and GWO has the best mean for F21, F22 and F23 as well. Finally, ALO has the best values of minimum, mean, median, and maximum in F17and F18, but gets the least mean, median, maximum, and standard deviation in F19. However, ALO has the best minimum and median for F21 and also scores the optimum for F22. Figures 3 and 4 show box plots and convergence rates for the results of some selected test functions.

### Comparisons with Some Advanced Variants of MRFO

In this subsection, the proposed MRFO-PSO is further evaluated through comparing its performance with some advanced variants of MRFO reported in the literature including modified MRFO (m-MRFO) [67], and MRFO and Gaussian mutation-based elephant herding optimization for global optimization (MGEHO) [54]. The results are recorded in Table 5 using the mean value of the fitness function along with the standard devotion (STD). It can be observed from the table that the proposed MRFO-PSO can exhibit very competitive results on most of the studied benchmark functions.

**Table 5:** The comparison of the MRFO-PSO and some advanced variants of MRFO

Fun	m-MRFO		MGEHO		MRFO-PSO	
	Mean	Std	Mean	Std	Mean	Std
F1	1.47E−270	0.00E + 00	0.00E + 00	0.00E + 00	**0.00E + 00**	**0.00E + 00**
F2	2.93E−135	9.31E−135	0.00E + 00	0.00E + 00	5.40E−323	**0.00E + 00**
F3	1.47E−263	0.00E + 00	6.68E−294	0.00E + 00	**0.00E + 00**	**0.00E + 00**
F4	4.51E−135	1.61E−134	0.00E + 00	0.00E + 00	**4.90E−324**	**0.00E + 00**
F5	2.40E + 01	4.10E−01	4.46E−297	0.00E + 00	**9.58E + 01**	1.12E + 00
F6	3.75E−07	2.53E−07	1.19E + 00	4.95 E00	**4.88E−01**	**3.51E−01**
F7	1.19E−04	1.06E−04	3.67E−03	7.03E−03	**2.28E−04**	**1.24E−04**
F8	− 7.51E + 03	6.62E + 02	0.00E + 00	0.00E + 00	− 2.37E−04	1.56E + 03
F9	0.00E + 00	0.00E + 00	7.01E−05	5.65E−05	**0.00E + 00**	**0.00E + 00**
F10	8.88E−16	0.00E + 00	0.00E + 00	0.00E + 00	**8.88E−16**	**0.00E + 00**
F11	0.00E + 00	0.00E + 00	− 1.26E04	1.41E + 01	**0.00E + 00**	**0.00E + 00**
F12	6.79E−09	4.71E−09	0.00E + 00	0.00E + 00	**3.46E−03**	**3.06E−03**
F13	2.02E + 00	1.37E + 00	8.88E−16	0.00E + 00	**9.89E + 00**	**1.95E−03**
F14	9.98E−01	1.37E−16	0.00E + 00	0.00E + 00	**9.98E−01**	**1.05E−16**
F15	3.38E−04	1.67E−04	9.02 E−4	3.29 E−3	**5.47E−04**	**4.65E−04**
F16	− 1.03E + 00	6.52E−16	2.12 E−3	8.10 E−3	**− 1.03E00**	**1.48E−16**
F17	3.98E−01	0.00E + 00	1.11E−293	0.00E + 00	**3.98E−01**	**0.00E + 00**
F18	3.00E + 00	1.57E−15	− 1.33E−15	0.00E + 00	**3.00E + 00**	2.34E−15
F19	− 3.86E + 00	2.67E−15	0.00E + 00	0.00E + 00	− **3.00E−01**	**6.90E−15**
F20	− 3.28E + 00	5.83E−02	0.00E + 00	0.00E + 00	− 3.26E00	**6.27E−02**
F21	− 9.98E + 00	9.31E−01	− 9.87E−01	2.28E−01	**− 6.07E00**	2.15E + 00
F22	− 1.00E + 01	1.35E + 00	0.00E + 00	0.00E + 00	**− 7.21E00**	2.74E + 00
F23	− 1.05E + 01	1.51E−15	0.00E + 00	0.00E + 00	− 7.70E00	3.01E + 00

Bold values best results among the compared optimizers are highlighted

### Lithium-Ion Battery

Rechargeable batteries have been used worldwide in numerous applications for instance: electric vehicles (EVs) [68], unmanned aerial vehicle (UAV), drones, flapping wing micro vehicles (FWMAVs) [69], aerospace missions, solar planets, wind power farms, electric sets, mobile phones, laptops, and power banks [70]. However, the several advantages of Li-ion batteries like long life, high cell voltage, low self-discharge rate as well as high energy density, encouraged engineers to utilize them in diverse systems. In contrast, there are some challenges such as increment of internal resistance, capacity deterioration due to degradation which will severely affect safety and distance vehicles can travel. In addition, there is no place for power supply failure in intensive care units, operations rooms in hospitals, giant firms' systems, and military training systems. Subsequently, it is a persistent necessity to estimate the 'State of Charge (SOC)' which determines the charge level by percentage as well as the remaining useful life (RUL) which defines the residual time required to subrogate the battery, so deficiency warning could be released prior the critical limit. To prohibit damages or calamitous collapses, periodic maintenance is a must. As a result, prognostication of battery main characteristics like SOC, RUL, current, and voltage is an urgent battery prognostics and health management problem which imposes itself in research scope [71]. Therefore, accurate dynamics modeling of batteries not only helps optimize design and manufacturing but also plays a crucial role in dismantling and re-usage exercised electrical vehicles (EV) batteries in implementations of the power grid. The more precise battery dynamics modeling is, the more sustainable the EV industry becomes. However, many models were proposed in the literature classified into three categories: electrochemical, analytical, and analog [72].

#### Tremblay's Model

**Fig. 5:**
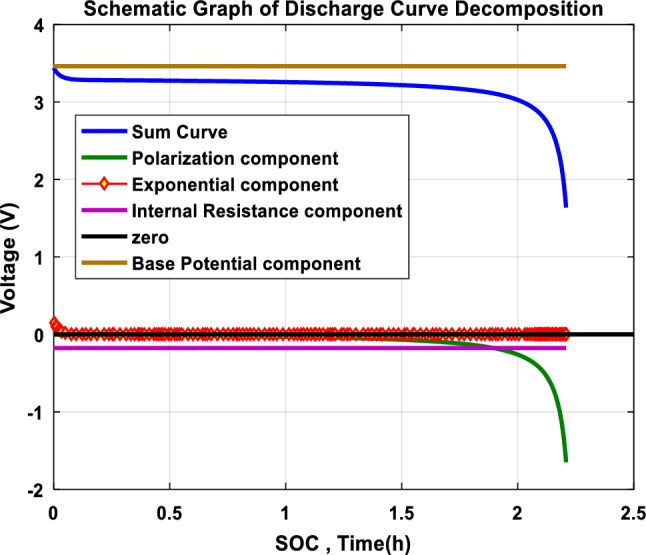
Schematic graph of the discharge curve

Tremblay's model [73] has been adopted by many researchers owing to its computational simplicity the reason why it operates exceedingly swift while running in software environments like MATLAB, and its efficacy during simulation, especially, EV applications. Nonetheless, Tremblay's model merges Li-ion battery dynamics, experiential and electrochemical simultaneously. The charge curve is analogous to the discharge curve. Howbeit, the discharge curve is formed by multiple zones shown in the schematic graph as in Fig. 5, the discharge voltage drops in the first sharp zone, thence it has an approximately fixed slope in the intermediate zone, and drops again sharply, contrariwise for charging. By electrochemical features of the Li-ion battery, the discharge curve is decomposed into four components: the base potential, exponential drop at the discharge start, a potential drop due to internal resistance, sharp potential drop at the discharge end, resulted from polarization. The discharge voltage can be estimated according to Eq. (14); otherwise, Eq. (15) describes the charge voltage [74]:

Discharge voltage:

14 $$V_{\tau } = E_{0} + \alpha \exp \left( { - \beta \Omega } \right) - I_{\tau } R - \frac{{CQ\Omega_{\tau } }}{{Q - \Omega_{\tau } }} - \frac{{CQI_{\tau }^{f} }}{{Q - \Omega_{\tau } }}.$$

Charge voltage:

15 $$V_{\tau } = E_{0} + \alpha \exp \left( { - \beta \Omega_{\tau } } \right) - I_{\tau } R - \frac{{CQ\Omega_{\tau } }}{{Q - \Omega_{t} }} - \frac{{CQI_{\tau }^{f} }}{{Q + 0.1\Omega_{\tau } }},$$

where exponential zone amplitude, exponential zone time constant inverse, and polarization voltage are represented by $\alpha , \beta ,\mathrm{ C}$ coefficients, respectively. While ${V}_{\tau }$ represents the voltage at time $\tau$,${E}_{0}$ is the base potential, and the internal resistance is$\mathrm{R}$, ${I}_{\tau }$ represents the discharge current at $\tau$ time; $Q$ is the nominal capacity; whereas ${\Omega }_{\tau }$ is the discharged capacity at $\tau$ time, which is derived from:${\Omega }_{\uptau }={\int }_{0}^{\uptau }{\mathrm{I}}_{\uptau }\mathrm{d\tau }$, since the current is constant so the ${\Omega }_{\uptau }={\mathrm{I}}_{\uptau }.\uptau$, ${\mathrm{I}}_{\uptau }^{\mathrm{f}}$ is the first-order step response usually called the filtered current at the time $\tau$ which is established as

16 $${I}_{\tau }^{f}={I}_{\tau }\cdot \left[1-\mathrm{exp}\left(-\frac{\tau }{{\tau }_{r}}\right)\right].$$

Consider $${\tau }_{r}$$ as the response time. Sequentially, there are four unknown parameters $$\alpha ,\beta ,\mathrm{ C},{E}_{0}$$ should be estimated; however, $$R,Q$$ are selected as nominal values by manufacturers, due to the human error in the three-key-point method as they depend on the personal perspectives and expertise, and the $$R,Q$$ should be also estimated to obtain more accurate simulation. Therefore, the control variable $$X=[\alpha ,\beta ,\mathrm{ C},{E}_{0},R,Q]$$ is the candidate solution for the parameter extraction problem of the Lithium battery dynamics model. Moreover, the objective function is taken as the residual sum of squares ($$RSS)$$

17 $$\mathrm{min}:\mathrm{ RSS}=\sum_{\mathrm{i}=1}^{\mathrm{m}} {\left[{\mathrm{V}}_{\mathrm{s}}^{\mathrm{i}}-{\mathrm{V}}_{\mathrm{c}}^{\mathrm{i}}\right]}^{2},$$

where ${V}_{s}^{i}$ is the discharge voltage sampled from the datasheet curve, ${V}_{c}^{i}$ is the calculated discharge voltage, and $m$ is the number of sampled points in the datasheet curve. In this study, we used the samples points extracted by Yong Wang and Lin Li uploaded in an Excel file on (http://binghamton.edu/seorl). For problem boundaries, we used their initial values ${X}_{initial}$ multiplied by 10 as the upper boundary, and 0 as the lower boundary for all cases [74]. In context, three cases are studied.

#### Case I

**Fig. 6:**
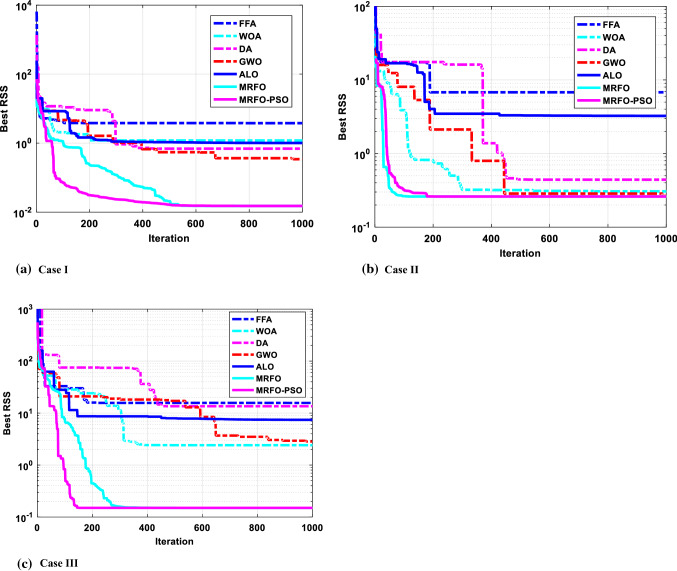
Convergence curve of $\mathrm{RSS}$ for the studied cases

**Fig. 7:**
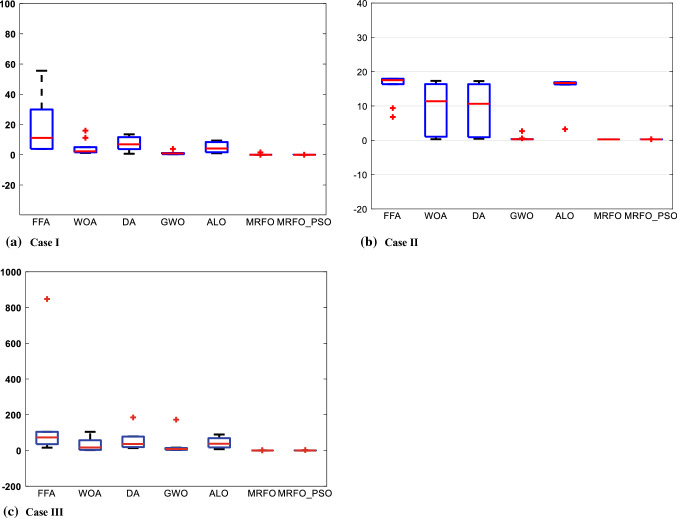
Box plots for the studied cases

**Fig. 8:**
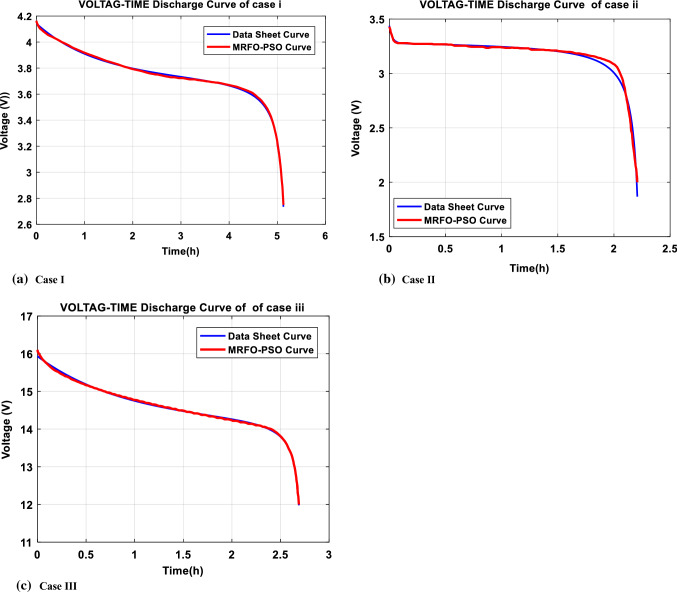
Discharge curve of extracted parameters by proposed MRFO-PSO for the studied cases

In this case, the Samsung Cylindrical ICR18650-22 lithium-ion rechargeable battery [75] is investigated with the upper and lower limits listed in Table 6. By implementing the proposed MRFO-PSO as well as the compared algorithms, we can obtain the results of the $RSS$ in terms of the statistical results as shown in Table 7. Furthermore, the optimal extracted parameters by the implemented algorithms corresponding to the best $RSS$ value are illuminated in Table 8. Based on the reported results, it can be observed that MRFO-PSO gets the minimum $RSS$ then GWO comes in the second order but DA gets the third. Additionally, MRFO-PSO comes first in terms of mean, maximum, and standard deviation but gets second in terms of median after MRFO. The convergence cures and box plots of the proposed MRFO-PSO as well as the compared algorithms are depicted in Figs. 6 and 7, respectively. Moreover, the estimated data obtained by MRFO-PSO and data sheet are compared in Fig. 8. Based on the figure, the estimated model exhibits a good agreement with the experimental data.

**Table 6:** The boundaries of studied cases

Case	Boundary	$\alpha$	$\beta$	$C$	${E}_{0}$	$R$	$Q$
Case I	Upper limit	3.9528	29.2162	37.724	0.0831	0.09	22.5
	Lower limit	0	0	0	0	0	0
Case II	Upper limit	1.3318	39.456	33.2597	0.0011	0.03	200
	Lower limit	0	0	0	0	0	0
Case III	Upper limit	13.7832	8.9532	147.523	0.0212	0.09	75
	Lower limit	0	0	0	0	0	0

**Table 7:** The statistical results of the different algorithms for the studied cases

Case		MRFO-PSO	MRFO	FFA	WOA	DA	GWO	ALO
Case I	Mean	0.016235	0.170724	90.99323	4.70351	7.308268	1.0944505	4.778585
	Min	0.015128	0.015128	3.79595	1.19448	0.689771	0.3423699	1.007829
	Median	0.016001	0.015128	11.15199	2.26922	6.867903	0.9118503	4.119789
	Max	0.018431	1.571092	757.9694	15.9253	13.49731	3.8770778	9.355362
	Std	0.001094	0.492039	234.9652	4.96859	4.660264	1.0286288	3.325728
Case II	Mean	0.270681	0.260513	1509.908	9.30681	8.970724	0.5584453	15.31753
	Min	0.260513	0.260513	6.783259	0.30657	0.441596	0.2672242	3.247749
	Median	0.260513	0.260513	17.52245	11.3762	10.62895	0.298084	16.69621
	Max	0.310986	0.260513	14,842.82	17.3089	17.2571	2.6692663	16.9655
	Std	0.021133	2.18E-15	4684.866	7.88719	7.025392	0.7469393	4.250027
Case III	Mean	0.421697	0.310146	2597.873	35.1644	55.93047	24.546672	43.47972
	Min	0.149853	0.149854	15.66367	2.41465	13.51091	2.8330533	7.420252
	Median	0.163295	0.149865	73.38813	16.8893	36.41602	8.3004532	37.58454
	Max	2.563543	1.666025	24,665.19	104.248	184.9011	172.67945	89.5566
	Std	0.754633	0.477183	7757.699	39.6977	52.52654	52.314716	31.75317

**Table 8:** The extracted parameters of the different algorithms for the studied cases

	Algorithm	$RSS$	$\alpha$	$\beta$	$C$	${E}_{0}$	$R$	$Q$
Case I	MRFO-PSO	0.015128	0.408138798	1.816450269	3.76149887	0.009370579	0.062567909	2.314153265
	MRFO	0.015128	0.408210849	1.814727487	3.773297582	0.009366075	0.089695572	2.314129007
	FFA	3.79595	3.9528	0.071449282	0.200178425	0.081721746	0.000111582	22.46093423
	WOA	1.19448	3.926476308	0	0	0.030688275	0	2.459480407
	DA	0.689771	0.34504181	19.0284594	3.877807207	0.019155345	0.017266594	2.375931164
	GWO	0.3423699	0.607953942	0.313907192	3.4136937	0.010858775	0.015435592	2.331619841
	ALO	1.007829	0.164669085	25.30505355	3.942980107	0.037869163	0.013518312	2.522679251
Case II	MRFO-PSO	0.260512901	0.160864758	3.863050155	3.31023996	0.0011	0.003277328	22.6269982
	MRFO	0.260512901	0.160864761	3.863050542	3.407918184	0.0011	0.01304515	22.6269982
	FFA	6.783258643	1.3318	10.72380686	3.238660431	0.0011	0	23.49264297
	WOA	0.306566872	0.219831245	39.45542764	3.337478509	0.001099984	0.006500495	22.63427802
	DA	0.441595806	0.153521702	0.379531854	3.251721849	0.000921267	0.000963452	22.54143344
	GWO	0.267224217	0.173290517	3.026364282	3.327238541	0.00108938	0.005261774	22.62390324
	ALO	3.247749115	0.991621827	16.62297171	3.299894051	0.00040399	0.011385273	22.26312555
Case III	MRFO-PSO	0.149853447	1.70387981	0.372380091	14.35588295	0.004737981	0.042818374	8.249542526
	MRFO	0.149853611	1.70378797	0.372500529	14.33802244	0.004739306	0.036796131	8.249592544
	FFA	15.66366727	13.74603659	0.021359455	2.040345018	0.021008118	0	74.37208551
	WOA	2.414653615	2.184801099	0.369542406	13.95881174	0.002862755	0.002389046	8.193942899
	DA	13.51090703	0.524766786	0.285550022	14.69115421	0.016788534	0.00275067	9.059359631
	GWO	2.833053252	3.673961949	0.06023017	12.14704898	0.005590857	0.089822861	8.329738685
	ALO	7.420252401	1.107769507	3.960014442	15.2361956	0.021196532	0.074839585	8.858287072

#### Case II

The second case is a prismatic cell produced by Tenergy manufacturer [76], and its boundary is in Table 8. The upper and lower limits are listed in Table 6. By conducting the proposed MRFO-PSO and the compared optimizers, we can achieve the results of the $RSS$ in terms of the statistical measures which are recorded in Table 7. In addition, the optimal estimated parameters by the implemented algorithms corresponding to the best $RSS$ value are presented in Table 8. Based on the reported results, it can be observed that MRFO-PSO can provide very competitive results regarding the $RSS$ value. The convergence behaviors and box plots of the proposed MRFO-PSO as well as the compared algorithms are depicted in Figs. 6 and 7, respectively. Moreover, the extracted data achieved by MRFO-PSO and data sheet are compared in Fig. 8. Based on the figure, the estimated model exhibits a good agreement with the experimental data.

#### Case III

The third case is UBBL03 (type LI-7) rechargeable battery cell produced by Ultralife manufacturer [77], and the upper and lower limits are listed in Table 6. By carrying out the proposed MRFO-PSO and the compared ones, we can obtain the optimized results of the $RSS$ in terms of the statistical results which are shown in Table 7. Furthermore, the optimal extracted parameters by the implemented algorithms corresponding to the best $RSS$ value are illuminated in Table 8. Based on the reported results, it can be observed that MRFO-PSO gets the minimum $RSS$ which is competitive with the MRFO, and can provide superior results over the compared ones. The convergence cures and box plots of the proposed MRFO-PSO as well as the compared algorithms are depicted in Figs. 6 and 7, respectively. Moreover, the identified parameters obtained by MRFO-PSO and data sheet are compared in Fig. 8. Based on the figure, it is noted that estimated parameters acquire a good agreement with the experimental data.

### Performance Assessment Based on Wilcoxon Test

The performance of the MRFO-PSO is further investigated to ensure that the obtained outcomes are not acquired by chance. In this sense, a non-parametric statistical test, named Wilcoxon signed-rank test, is applied [78] are performed. The Wilcoxon’s test is applied on the resulted mean values of the benchmark functions.

The Wilcoxon test is presented to illustrate the statistical significant difference among the obtained results by proposed MRFO-PSO algorithm and other peers. The outcomes of Wilcoxon’s test are recorded in Table 9. The rank ${R}^{+}$ values have larger values than the opposite rank ${R}^{-}$, which means that all tests reject the null hypothesis. Moreover, the p value is smaller than the significance level (${\alpha }_{sig}=0.05)$ for most cases, which ensured the superior results of MRFO-PSO over the compared ones. From Table 9, it can be noted that the results of MRFO-PSO is not significant ($\approx$) to those obtained by MGEHO as the significance level is greater than $0.05$. Furthermore, the Wilcoxon’s test is applied on the studied cases of the lithium-ion battery model by carrying out each algorithm for some different runs. The best results of all runs are employed as the samples for Wilcoxon test, and then, the results of this are reported in Table 10. From Table 10, it can be clearly observed the MRFO-PSO is very competitive with MRFO and outperforms the other ones.

**Table 9:** Wilcoxon’s test for the reported results in Tables 4 and 5

Compared methods		$p$ Value	Solution evaluations		
Proposed	Competitors		${R}^{+}$	${R}^{-}$	Winner
MRFO-PSO	FFA	0.00001	253	0	MRFO-PSO
MRFO-PSO	WOA	0.02444	114	39	MRFO-PSO
MRFO-PSO	DA	0.00001	231	0	MRFO-PSO
MRFO-PSO	GWO	0.01078	129	42	MRFO-PSO
MRFO-PSO	ALO	0.00034	184	6	MRFO-PSO
MRFO-PSO	MRFO	0.0151	58	8	MRFO-PSO
MRFO-PSO	m-MRFO	0.00374	97	23	MRFO-PSO
MRFO-PSO	MGEHO	0.11642	78	93	$\approx$

**Table 10:** Wilcoxon’s test for the studied cases of the lithium-ion battery

Compared methods			Solution evaluations		
Proposed	Competitors	$p$ Value	${R}^{+}$	${R}^{-}$	Winner
MRFO-PSO	FFA	0.00001	465	0	MRFO-PSO
MRFO-PSO	WOA	0.00001	465	0	MRFO-PSO
MRFO-PSO	DA	0.00001	465	0	MRFO-PSO
MRFO-PSO	GWO	0.00001	465	0	MRFO-PSO
MRFO-PSO	ALO	0.00001	465	0	MRFO-PSO
MRFO-PSO	MRFO	0.02202	197	56	MRFO-PSO

As depicted in Tables 9 and 10, in most tasks, the recorded $p$ value is far less than 0.05, which affirms that MRFO-PSO has stronger significance.

## Conclusion and future work

A new hybrid Manta ray foraging optimization (MRFO) with particle swarm optimization (PSO), named MRFO-PSO, was presented for further promoting the harmony among the inclusive exploration and confined exploitation abilities while dealing with optimization tasks. The MRFO-PSO was conducted and validated on a well-studied set of benchmark problems along with the comparisons with some optimization methods. The experimental results were made through evaluating some statistical measures and non-parametric tests which have demonstrated that the MRFO-PSO provides competitive and progressive solutions compared with other competitors. In addition, the applicability of MRFO-PSO is performed to estimate the Tremblay's model of the lithium-ion battery. The final experimental results illustrate that the MRFO-PSO can contribute powerful assistance for the lithium-ion battery and it has the potential to be very fruitful in dealing with more practical tasks with complicated search spaces as well. The major contributions regarding the presented work are

- 1. The proposed MRFO-PSO enhances the convergence rate and population diversity of the original MRFO by achieving the global solution after a few iterations.
- 2. MRFO-PSO confirmed its effectiveness by the comparison with other optimization methodologies while dealing with large-scale benchmark functions of different complexities.
- 3. MRFO-PSO has affirmed its applicability by estimating the parameters of the lithium-ion battery.

### Future Work

The increasing popularity of electric vehicles highlights the importance of the study of lithium batteries for electric vehicles, The lithium battery used in electric vehicles is a very large battery pack, and the testing of the SOC and SOH of the whole battery pack requires the support of experimental equipment, to overcome these issues, we will endeavor to include the volume of the battery pack in the future model, as well as built the future failure time for the battery pack.

## Data Availability

Not applicable.

## Appendix

See Table 11

**Table 11:** The abbreviations and symbols used in this paper

NLP	Nonlinear problems
MRFO	Manta ray foraging optimization
PSO	Particle swarm optimization
MRFO-PSO	Hybrid MRFO with PSO
EHO	Elephant herding optimization
OBL	Opposition-based learning
CT	Computerized tomography
Covid-19	Coronavirus disease 2019
SVM	Support vector machine
ECG	Electrocardiogram
CNN	Convolutional neural network
GA	Genetic algorithm
MR	Magnetorheological
GMRFO	Genetic Manta ray foraging optimization
QMRFO	Quantum Manta ray foraging optimization Algorithm
PV GBO MRFO–GBO EED	Solar photovoltaic Gradient-based optimizer Integrated MRFO with GBO Economic emission dispatch
${F}_{i}(x)$	Fitness function
$n$	No. of dimensions
$x$	Candidate solution
$i\mathrm{th}$	Order of the variable
${l}_{i}$	Lower limit
${u}_{i}$	Upper limit
${\mathfrak{R}}^{n}$	Design space
${x}_{i}^{t}$	The *i*th individual's position at the iteration $t$
$r$	Random vector belong to [0, 1]
$a$	Weighting function
${x}_{b}$	The best position where plankton is concentrated
$\varpi$	Random number within the range of [0, 1]
$\mathrm{B}$	Weight coefficient
$T$	Total number of iterations
${r}_{1}$	Random number
${x}_{\text{rand}}$	Random position placed indiscriminately in the search space
$\psi$	Somersault factor
${r}_{2},{r}_{3}$	Random values within [0, 1] range
${v}_{i}$	Bird (particle) velocity
${x}_{i}^{t+1}$	The particle position at the next iteration $t+1$
$\gamma$	Best position of the whole birds
${P}_{i}$	The best position bird had
$\omega$	Weighting function,
${\vartheta }_{i}^{t+1}$	The individual position reached by its velocity
${v}_{i}^{t+1}$	Velocity of Manta ray individual
${\Upsilon }_{1}$	Best position reached by Manta ray individual 's velocity obtained so far
${\Upsilon }_{2}$	Second best individual position reached in the last iteration
$\alpha$	Exponential zone amplitude
$\beta$	Exponential zone time constant inverse
$C$	Polarization voltage
${V}_{\tau }$	The voltage at time $\tau$
${E}_{0}$	The base potential
$R$	The internal Resistance
${I}_{\tau }$	The discharge current at $\tau$ time
$Q$	The nominal capacity
${\Omega }_{\tau }$	The discharged capacity at $\tau$ time
${I}_{\tau }^{f}$	The first-order step response usually called the filtered current at time $\tau$
${\tau }_{r}$	Response time
$RSS$	The residual sum of squares
${V}_{s}^{i}$	The discharge voltage sampled from datasheet curve
${V}_{c}^{i}$	The calculated discharge voltage
$m$	The number of sampled points in datasheet curve
${\mathrm{X}}_{\text{initial}}$	Initial values of Tremblay's model
${\alpha }_{sig}$	Significance level
${R}^{-}$	Sum of pos. ranks
${R}^{+}$	The sum of neg. ranks
